# Prediction model of no-response before the first transarterial chemoembolization for hepatocellular carcinoma: TACF score

**DOI:** 10.1007/s12672-023-00803-2

**Published:** 2023-10-17

**Authors:** Jia-Wei Zhong, Dan-Dan Nie, Ji-Lan Huang, Rong-Guang Luo, Qing-He Cheng, Qiao-Ting Du, Gui-Hai Guo, Liang-Liang Bai, Xue-Yun Guo, Yan Chen, Si-Hai Chen

**Affiliations:** 1 Department of Gastroenterology, Digestive Disease Hospital, The First Affiliated Hospital of Nanchang University, Nanchang, Jiangxi China; 2 Medical Imaging Department, The First Affiliated Hospital of Nanchang University, Nanchang, Jiangxi China; 3 Department of Interventional Medicine, The First Affiliated Hospital of Nanchang University, Nanchang, Jiangxi China; 4 Department of Gastroenterology, Fengcheng People’s Hospital, Fengcheng, Jiangxi China; 5 Postdoctoral Innovation Practice Base, The First Affiliated Hospital of Nanchang University, Nanchang, People’s Republic of China

**Keywords:** Hepatocellular carcinoma, Transarterial chemoembolization, First response, Individual prediction

## Abstract

Previous clinic models for patients with hepatocellular carcinoma (HCC) receiving transarterial chemoembolization (TACE) mainly focused on the overall survival, whereas a simple-to-use tool for predicting the response to the first TACE and the management of risk classification before TACE are lacking. Our aim was to develop a scoring system calculated manually for these patients. A total of 437 patients with hepatocellular carcinoma (HCC) who underwent TACE treatment were carefully selected for analysis. They were then randomly divided into two groups: a training group comprising 350 patients and a validation group comprising 77 patients. Furthermore, 45 HCC patients who had recently undergone TACE treatment been included in the study to validate the model’s efficacy and applicability. The factors selected for the predictive model were comprehensively based on the results of the LASSO, univariate and multivariate logistic regression analyses. The discrimination, calibration ability and clinic utility of models were evaluated in both the training and validation groups. A prediction model incorporated 3 objective imaging characteristics and 2 indicators of liver function. The model showed good discrimination, with AUROCs of 0.735, 0.706 and 0.884 and in the training group and validation groups, and good calibration. The model classified the patients into three groups based on the calculated score, including low risk, median risk and high-risk groups, with rates of no response to TACE of 26.3%, 40.2% and 76.8%, respectively. We derived and validated a model for predicting the response of patients with HCC before receiving the first TACE that had adequate performance and utility. This model may be a useful and layered management tool for patients with HCC undergoing TACE.

**Supplementary Information:**

The online version contains supplementary material available at 10.1007/s12672-023-00803-2.

## Introduction

Transarterial chemoembolization (TACE) is universally acknowledged as an effective treatment for patients with intermediate and advanced hepatocellular carcinoma (HCC) [1–3] and definitely prolongs the survival of those patients [1, 4]. However, due to the heterogeneity of patients with HCC, which contributes to a heterogeneous response to TACE [5–7], approximately half of the patients receiving TACE did not obtain an objective response [8]. The accurate identification of patients with a high probability of nonresponse to the first TACE is crucial for treatment strategy decisions [9]. Therefore, a convenient and effective way to screen out those patients before TACE might light on decision making.

Based on the different tumor characteristics and statuses of individual patients with HCC, selecting the most appropriate treatment for every patient with HCC is the “ideal” treatment procedure. The establishment of clinical prediction models has been widely used to customize personalized treatment [10, 11]. Some individualized prediction models for patients receiving TACE have been developed to predict the prognosis of those patients and include the six-and-twelve score [12], HAP score [13] and SNACOR model [14]. However, those models did not focus on the treatment response of the first TACE, but the prediction of overall survival (OS). Recently, an increasing number of predictive models have focused on the first response to TACE. Fundamental prognostic models of radiomics based on ultrasound [15], computed tomography (CT) [16, 17], magnetic resonance imaging (MRI) [18, 19] and digital subtraction angiography (DSA) [20] for predicting the response to the first TACE showed adequate performance. However, only the texture of the tumor was included in those models, and personal liver function was excluded, which might overlook some vital clinical information [3]. A machine-learning model that combined imaging features and liver function, provided an accurate prediction of the first response to TACE, but the complicated computational formula limited its practicability in the clinic [21]. Thus, the development of a simple-to-use, accurate and customized model for predicting the initial response to TACE among patients with HCC is desired.

In this study, we aimed to drive and validate the personalized model, which incorporated both objective imaging characteristics and serological testing, for predicting the probability of a response to the first TACE, and it can be simply and accurately used to guide clinical decisions.

## Patients and methods

### Study design and patient eligibility

This study was designed according to the Transparent Reporting of a Multivariable Prediction Model for Individual Prognosis or Diagnosis (TRIPOD) [22] to establish a reliable and feasible prediction model for calculating the nonresponse probability of TACE [23]. The checklist of items is shown in Supplemental Table 1.

**Fig. 1:**
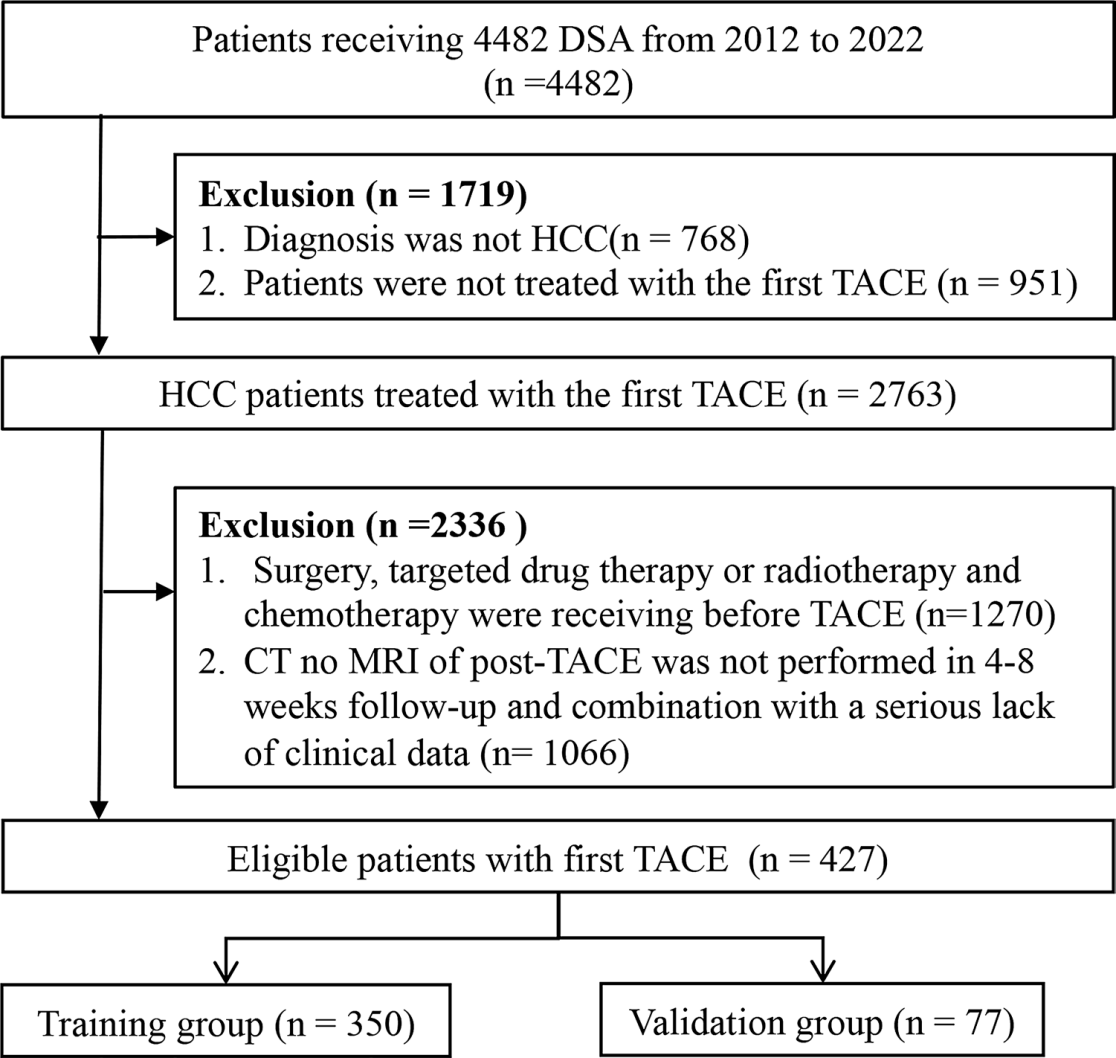
Flow chart of the selection process. *DSA* digital subtraction angiography, *HCC* hepatocellular carcinoma, *TACE* transarterial chemoembolization

This retrospective study consisted of consecutive adult patients who underwent DSA between January 2012 and January 2022 at the Department of Gastroenterology or Interventional Surgery, the First Affiliated Hospital of Nanchang University. A total of 4482 consecutive patients receiving DSA were retrospectively screened. Our target patients are HCC patients who have not received any before TACE. Patients were excluded if (1) the diagnosis was not HCC, (2) patients were received TACE, surgery, targeted drug therapy or radiotherapy and chemotherapy before hospitalization, (3) CT or MRI was not performed at the 4–8-week follow-up after TACE, or (4) a serious lack of clinical data existed. The flow chart of the selection process is shown in Fig. 1. Finally, 437 patients with unresectable who underwent a first TACE were recruited for analysis. Besides, for enhanced model validation and to ascertain its stability, we gathered data from patients who underwent first TACE between February 2022 and July 2023.

Patients with BCLC B and C stage undergo conventional TACE treatment, including those with multiple lesions, portal vein invasion, or extrahepatic spread, which based on the Barcelona Clinic Liver Cancer (BCLC) stage [24], strictly.

To ensure an ample sample size for rigorous model validation, effectively achieving our goal, the total patients were randomly divided into two groups, including training (70%) and validation groups (30%), which is supported by TRPOD standards [22] and seven steps for development and an ABCD for validation [25].

The clinical baseline characteristics were collected before the first TACE, including age, sex, HBV infection status, the status of cirrhosis, diabetes, rupture and bleeding of HCC, hemoglobin (Hb) level, red blood count (RBC), white blood cell count (WBC), neutrophil count, lymphocyte count, platelet (PLT) count, albumin (ALB), globulin (GLB), total bilirubin (TBIL), direct bilirubin (DBIL), aspartate aminotransferase (AST), alanine aminotransferase (ALT), potassium (K^+^), prothrombin time (PT), international normalized ratio (INR) and a-fetoprotein (AFP) levels. The objective imaging characteristics of the tumor, including the maximum diameter of the lesion, hepatic arteriovenous fistula (HAVF), portal vein tumor thrombus (PVTT) and the integrality of the tumor capsule, were independently screened before the first TACE by two radiologists. When the radiologists disagreed on the results of objective imaging characteristics, the two radiologists discussed them together until they reached an agreement. The independent assessment of these objective radiological biomarkers by the two radiologists showed good consistency. However, there was inconsistency in HAVF, PVTT, and the integrality of the tumor capsule in fewer than 10 patients. All clinical data were obtained within a week before TACE. MRI or CT was performed within 4–6 weeks after TACE to obtain the first treatment response. The response to TACE was strictly evaluated by one gastroenterologist experienced with TACE and two radiologists independently, which was based on the modified Response Evaluation Criteria in Solid Tumors (mRECIST) [26]. According to the response to TACE, progressive disease (PD) and stable disease (SD) were considered nonresponses to TACE, and a complete response (CR) and partial response (PR) were considered responses to TACE.

### Diagnosis and staging of HCC

The diagnosis of HCC was based on the guideline proposed by the National Health Commission of the People’s Republic of China [27]. According to the guideline, HCC was diagnosed if patients had one or more risk factors: (1) lesions less than 2 cm in diameter and two typical imaging manifestations, including ultrasound, enhanced CT, MRI and DSA; (2) lesions more than 2 cm in diameter with one typical imaging manifestation; and (3) one typical imaging manifestation, and an AFP level ≥ 400 μg/L.

The BCLC staging [24], albumin–bilirubin (ALBI) scoring [28] and Child–Pugh (C–P) grade [29] were also evaluated. The classification of the ALBI score was as follows: ALBI grade 1 ≤ − 2.60, ALBI grade 2 between − 2.6 and − 1.39, ALBI grade 3 > − 1.39.

### Treatment procedures

The femoral artery approach was routinely chosen. The patient was placed in a supine position, disinfected, covered with a towel, and subjected to local infiltration anesthesia. The Seldinger technique was used to percutaneously puncture the femoral artery, insert the catheter sheath, and insert the catheter into the celiac trunk artery or common hepatic artery for angiography. If vascular scarcity or absence is identified in a specific liver region, it suggests the presence of collateral circulation that supports tumor growth. Therefore, further investigation of corresponding arteries is necessary, including the superior mesenteric artery, intercostal arteries below the ribs, subdiaphragmatic artery, adrenal inferior artery branching from the renal artery, adrenal middle artery, left gastric artery, lumbar artery, internal mammary artery, among others. Conventional catheters or microcatheters were used to superselect the tumor feeding artery, and cisplatin, oxaliplatin, lobaplatin, and fluorouracil were diluted 0.9% saline solution or 5% glucose solution and slowly injected into the target artery. After arteriovenous fistula was excluded by angiography, an iodized oil emulsion was injected. The ratio of iodized oil (1–20 mL) and aqueous pirarubicin solution was 2:1, and the iodized oil emulsion was prepared after mixing. The iodized oil emulsion was slowly injected into the target artery. If arteriovenous fistulas were present, the fistulas were embolized with coils or gelatin sponges before the iodized oil emulsion was injected. After the injection of the iodized oil emulsion, the blood flow was slowed, and the injection embolization was performed with microspheres or gelatin sponge particles. Further angiography confirmed that the end point of embolism had been reached. If the patient was considered for emergency embolization treatment of rupture and hemorrhage of liver cancer, the infusion of chemotherapy drugs was not administered. The embolization endpoint is achieved when the supplying artery appears as a “dry twig”, indicating successful embolization of small tumor-feeding arteries while preserving the patency of liver segment or lobar arteries. This approach facilitates subsequent embolization treatments. Abdominal CT or MRI was performed 4–8 weeks after TACE to evaluate the treatment effect, and the next TACE was performed if necessary.

### Statistical analysis

Based on whether the data fit a normal distribution, quantitative variables for patients at baseline are presented as the medians with interquartile ranges [IQRs] or the means with standard deviations. Categorical variables for patients at baseline and tumor characteristics measured using CT are presented as counts and percentages. The T test or nonparametric Mann‒Whitney U test and Chi square test or Fisher’s exact test were used to compare quantitative or categorical variables between the two groups, respectively. The information of missing data was showed in Supplemental Figure 2, including overall cohorts, development and training data. In the development data, a total of 337 patients with complete serum and imaging data were available for lasso regression. Among them, only 13 patients had missing data, with ALB, one of the predictive indicators, being missing in only 2 cases. Given the limited number of missing values, we opted to exclude these instances while selecting the predictive factors (Supplemental Figure 4).

Candidate risk factors for a nonresponse to TACE in patients with HCC were evaluated using a univariate logistic regression analysis and least absolute shrinkage and selection operator (LASSO) logistic regression analysis [30]. Potential variables with P < 0.05 in the univariate logistic regression analysis or a coefficient > 0 were selected for the multivariate logistic regression analysis to identify the variables to construct the models. In the development group, there were a total of 337 patients with complete serum and imaging data available for lasso regression. Only 13 patients had missing data, and among them, only 2 patients had missing values for ALB, one of the predictive indicators. Generalized variance inflation factor (GVIF) and correlation analyses were performed to avoid multicollinearity in the construction of models. The GVIF and correlation coefficient values were lower than 5 [31] and 0.25 respectively, indicating the probability of multicollinearity among the variables was low. The candidate models of the discrimination and calibration abilities were generated by constructing receiver operating characteristic curves (ROCs) and calibration plots, respectively. The best model was selected using the Hosmer‒Lemeshow test, Akaike information criterion (AIC), area under the receiver operating characteristic (AUROC) curve, calibration plot slope and decision curve analysis (DCA) [32].

We evaluated approximately 10 variables in the final model. Based on the rule of 10 events per variable fitting the logistic regression model [33], the probability of objective response to TACE was approximately 52.5% [8]. Thus, at least 200 eligible patients were included in the training group. In fact, 154 patients in the training group did not respond to TACE, which exceeded the expected number. Therefore, this study had an adequate sample size.

Patients with missing values were included in the baseline analysis but excluded from the correlation analysis, LASSO and logistic regression analyses. A P value less than 0.05 was considered significant. An analysis of trends was performed using the Cochran–Armitage trend test. All statistical analyses were performed using R software (version 4.2.1). The packages dplyr, tableone, rms, PredictABEL, nomogramFormula, corrplot, DescTools, ggpolt2, VIM and ggDCA were used to clear, analyze, and visualize the data.

## Results

### Baseline characteristics

A total of 427 eligible patients were selected, of which 350 patients were assigned to the training group. The demographics and clinical characteristics are shown in Table 1. The demographics, objective imaging characteristics and HCC staging were not different between the training and validation groups, except for K^+^. The nonresponse rates to the first TACE in the two groups were 44% (154 events) and 51.9% (40 events). The demographics and clinical characteristics of 45 HCC patients who had recently undergone TACE treatment are shown in Supplemental Table 6.

**Table 1:** Demographics and clinical characteristics of eligible patients with TACE

	All patients (n = 427)	Training group (n = 350)	Validation group (n = 77)	P value
Sex				0.229
Male	367 (85.9)	297 (84.9)	70 (90.9)	
Female	60 (14.1)	53 (15.1)	7 (9.1)	
Age	55.00 [46.00, 64.00]	54.00 [45.00, 63.00]	59.00 [49.00, 67.00]	0.056
Etiology				0.333
Other	56 (13.1)	49 (14.0)	7 (9.1)	
HBV	371 (86.9)	301 (86.0)	70 (90.9)	
Diabetes				1.00
No	404 (94.6)	331 (94.6)	73 (94.8)	
Yes	23 (5.4)	19 (5.4)	4 (5.2)	
Cirrhosis				0.89
No	216 (50.6)	176 (50.3)	40 (51.9)	
Yes	211 (49.4)	174 (49.7)	37 (48.1)	
Hb (g/L)	128.00 [115.00, 143.00]	128.0 [115.0, 142.0]	132.0 [117.0, 145.0]	0.552
RBC (×10^12^/L)	4.21 [3.79, 4.72]	4.20 [3.76, 4.77]	4.25 [3.87, 4.69]	0.853
WBC (×10^9^/L)	5.26 [4.00, 7.13]	5.30 [4.01, 7.29]	5.10 [3.95, 6.69]	0.311
The count of neutrophils (×10^9^/L)	3.49 [2.44, 5.07]	3.51 [2.42, 5.19]	3.47 [2.44, 4.39]	0.309
Lymphocyte count (×10^9^/L)	1.08 [0.79, 1.44]	1.06 [0.78, 1.45]	1.11 [0.85, 1.41]	0.347
PLT (×10^9^/L)	146.0 [86.00, 210.0]	147.00 [89.0, 207.75]	138.00 [83.00, 221.00]	0.943
ALB (g/L)	37.20 [33.60, 41.00]	37.05 [33.38, 40.92]	37.40 [34.80, 41.70]	0.459
GLB (g/L)	29.80 [26.10, 33.60]	29.90 [26.30, 33.90]	29.30 [25.30, 32.30]	0.352
TBIL (μmol/L)	15.30 [10.40, 20.90]	15.30 [10.53, 20.58]	15.20 [9.70, 21.90]	0.897
DBIL (μmol/L)	5.60 [3.60, 8.80]	5.70 [3.70, 8.80]	5.20 [3.50, 8.00]	0.505
AST (U/L)	36.00 [25.00, 56.00]	37.50 [25.00, 57.75]	35.00 [25.00, 50.00]	0.362
ALT (U/L)	146.00 [86.00, 210.00]	147.00 [89.0, 207.75]	138.00 [83.00, 221.00]	0.759
K^+^ (mmol/L)	4.02 [3.74, 4.36]	3.99 [3.70, 4.35]	4.20 [3.90, 4.44]	0.003
PT (s)	12.40 [11.70, 13.40]	12.40 [11.70, 13.50]	12.20 [11.60, 13.20]	0.273
INR	1.09 [1.03, 1.19]	1.10 [1.03, 1.20]	1.09 [1.01, 1.16]	0.248
Ascites				0.219
None	290 (67.9)	232 (66.3)	58 (75.3)	
Mild or moderate	104 (24.4)	88 (25.1)	16 (20.8)	
Severe	33 (7.7)	30 (8.6)	3 (3.9)	
AFP (ng/mL)				0.847
< 40	139 (32.6)	116 (33.2)	23 (29.9)	
≥ 40 and < 400	81 (19.0)	66 (18.9)	15 (19.5)	
≥ 400	206 (48.4)	167 (47.9)	39 (50.6)	
TACE response				0.254
Responders	233 (54.6)	196 (56.0)	37 (48.1)	
Non-responder	194 (45.4)	154 (44.0)	40 (51.9)	
Objective imaging characteristics				
Tumor size (cm)				0.776
≤ 5	133 (31.1)	107 (30.6)	26 (33.8)	
> 5 and ≤ 10	176 (41.2)	144 (41.1)	32 (41.6)	
> 10	118 (27.6)	99 (28.3)	19 (24.7)	
Numbers of tumor				1
Single	214 (50.1)	175 (50.0%)	39 (50.6)	
Multiple	213 (49.9)	175 (50%)	38 (49.4)	
HAVF				0.138
No	389 (91)	315 (90.0)	74 (96.1)	
Yes	38 (8.9)	35 (10)	3 (3.9)	
PVTT				1
No	293 (68.6)	240 (68.6)	53 (68.8)	
Yes	134 (31.4)	110 (31.4)	24 (31.2)	
Integrality of tumor capsule				0.762
Yes	97 (22.7)	78 (22.3)	19 (24.7)	
No	330 (77.3)	272 (77.7)	58 (75.3)	
Rupture and bleeding of HCC				0.832
No	394 (92.3)	322 (92.0)	72 (93.5)	
Yes	33 (7.7)	28 (8.0)	5 (6.5)	
HCC staging				
C–P class				0.121
A	308 (73.9)	246 (72.1)	62 (81.6)	
B or C	109 (26.1)	95 (27.9)	14 (18.4)	
ALBI grade				0.964
I	153 (36.0)	126 (36.2)	27 (35.1)	
II	272 (64.0)	222 (63.8)	50 (64.9)	
BCLC stage				0.826
B stage	119 (28.2)	94 (27.8)	23 (29.9)	
C stage	303 (71.8)	249 (72.2)	54 (70.1)	

*TACE* transarterial chemoembolization, *Hb* hemoglobin, *RBC* red blood count, *WBC* white blood cell count, *PLT* platelet, *ALB* albumin, *GLB* globulin, *TBIL* total bilirubin, *DBIL* direct bilirubin, *AST* aspartate aminotransferase, *ALT* alanine aminotransferase, *K*^*+*^ potassium, *PT* prothrombin time, *INR* international normalized ratio, *AFP* α-fetoprotein, *HAVF* hepatic arteriovenous fistula, *PVTT* portal vein tumor thrombus, *BCLC* Barcelona Clinic Liver Cancer, *ALBI* albumin–bilirubin, *C–P* Child–Pugh

### Candidate predictive factors for a nonresponse to the first TACE

Based on the TRIPOD guideline, a comprehensive literature review of TACE was performed. The five objective imaging characteristics, including the maximum tumor size, numbers of tumors, HAVF, PVTT, and integrality of the tumor capsule, were potential predictive factors of nonresponse to the first TACE. We also identified that ALB, TBIL, DBIL, AST and ALT levels were potentially associated with nonresponse to the first TACE. In addition, other serum indices and clinical characteristics were also selected for LASSO and univariate logistic regression analyses.

**Fig. 2:**
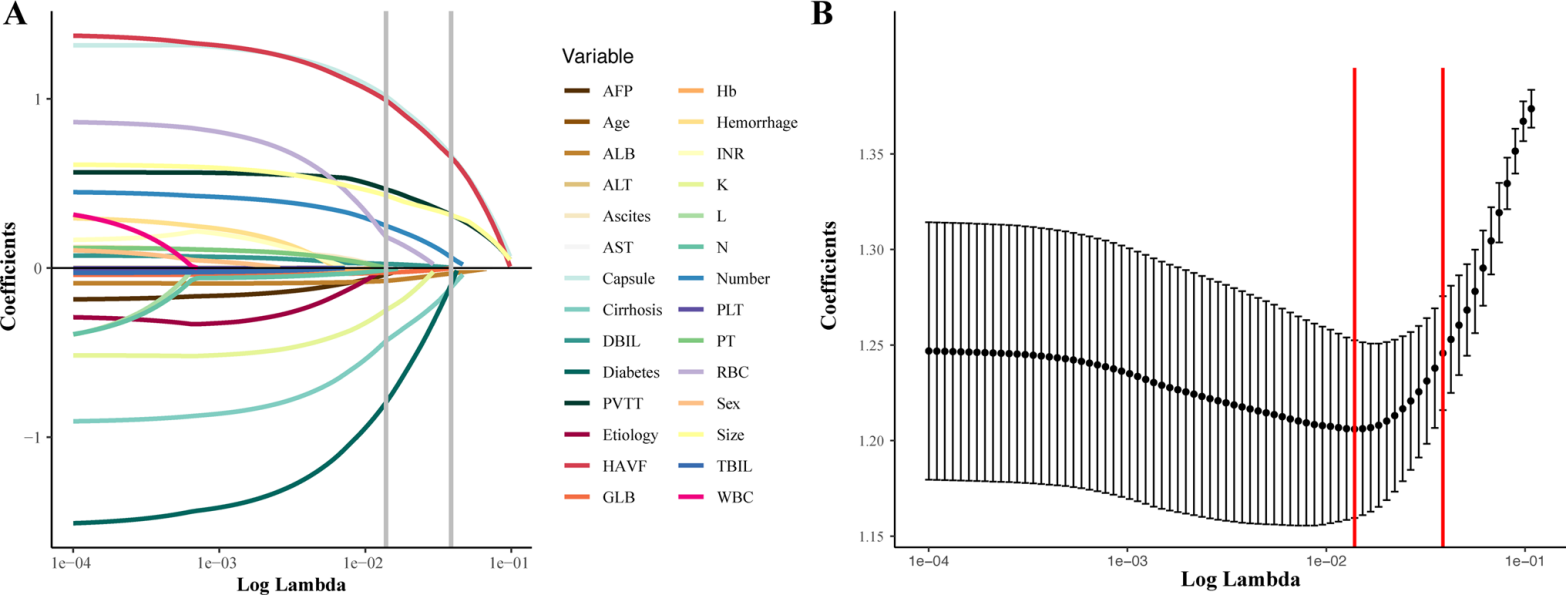
Texture feature selection using LASSO regression. **A**, **B** Showed the shrinkage process of coefficients. *LASSO* least absolute shrinkage and selection operator, *RBC* red blood count, *WBC* white blood cell count, *PLT* lymphocyte count, platelet; *ALB* albumin, *GLB* globulin, *TBIL* total bilirubin, *DBIL* direct bilirubin, *AST* aspartate aminotransferase, *ALT* alanine aminotransferase, *K*^*+*^ potassium, *PT* prothrombin time, *L* lymphocyte count, *N* neutrophil count, *INR* international normalized ratio, *AFP* a-fetoprotein, *HAVF* hepatic arteriovenous fistula, *PVTT* portal vein tumor thrombus, *Number* numbers of tumor, *Size* maximum diameter of tumor

In the LASSO logistic regression analysis, 28 features were reduced to 11 candidate predictors with nonzero coefficients, including numbers of tumors, integrality of the tumor capsule, HAVF, diabetes, cirrhosis, ALB, GLB, DBIL, AST and PVTT (Fig. 2A, B). The results from the univariate logistic regression analysis are shown in Table 2. In the univariate logistic regression analysis, cirrhosis, ALB, AST, max tumor size, numbers of tumors, HAVF, PVTT and integrality of the tumor capsule were potential predictive factors of nonresponse to the first TACE, whereas the DBIL level showed borderline statistical significance (P value = 0.06). The status of cirrhosis might be associated with ALB, AST and DBIL levels, which might contribute to multicollinearity. Finally, AST, ALB, DBIL, maximum tumor size, numbers of tumors, HAVF, PVTT and integrality of the tumor capsule were selected for the multivariate logistic regression analysis (Table 3).

**Table 2:** Univariate analysis of predictive factors for non-response of TACE

Variable	Univariate analysis	
	OR (95% CI)	P value
Sex		
Male	Reference	
Female	0.887 (0.491, 1.604)	0.692
Age	0.987 (0.971, 1.004)	0.139
Etiology		
Other	Reference	
HBV	0.871 (0.475, 1.596)	0.655
Diabetes		
No	Reference	
Yes	0.871 (0.475, 1.596)	0.655
Cirrhosis		
No	Reference	
Yes	0.611 (0.399, 0.935)	0.023*
Hb (g/L)	0.9995 (0.9905, 1.0085)	0.911
RBC (×10^12^/L)	1.224 (0.946, 1.583)	0.124
WBC (×10^9^/L)	1.017 (0.951, 1.089)	0.618
The count of neutrophils (×10^9^/L)	1.017 (0.943, 1.098)	0.657
lymphocyte count (×10^9^/L)	0.929 (0.62, 1.392)	0.720
PLT (×10^9^/L)	1.00 (0.9994, 1.0039)	0.161
ALB (g/L)	0.942 (0.903, 0.983)	0.006*
GLB (g/L)	0.980 (0.945, 1.015)	0.258
TBIL (μmol/L)	1.0069 (0.9883, 1.0258)	0.469
DBIL (μmol/L)	1.032 (0.999, 1.067)	0.06
AST (U/L)	1.0061 (1.0019, 1.0103)	0.005^*^
ALT (U/L)	1.0032 (0.999, 1.0074)	0.135
K^+^ (mmol/L)	0.899 (0.572,1.415)	0.646
PT (s)	1.027 (0.902, 1.169)	0.69
INR	1.416 (0.341, 5.887)	0.632
Ascites		
None	Reference	
Mild or moderate	1.172 (0.716, 1.919)	0.5283
Severe	1.176 (0.548, 2.521)	0.6779
AFP (ng/mL)		
< 40	Reference	
≥ 40 and < 400	1.172 (0.716, 1.919)	0.5283
≥ 400	1.176 (0.548, 2.521)	0.6779
Tumor size (cm)		
≤ 5	Reference	
> 5 and ≤ 10	2.133 (1.255, 3.627)	0.0051**
> 10	3.193 (1.794, 5.683)	< 0.001**
Numbers of tumor		
Single	Reference	
Multiple	1.836 (1.198, 2.815)	0.005**
AVF		
No	Reference	
Yes	4.22 (1.914, 9.305)	< 0.001**
PVTT		
No	Reference	
Yes	2.451 (1.545, 3.888)	< 0.001**
Integrality of tumor capsule		
Yes	Reference	
No	3.06 (1.732, 5.405)	< 0.001**
Rupture and bleeding of HCC		
No	Reference	
Yes	1.519 (0.7, 3.296)	0.29

*TACE* transarterial chemoembolization, *Hb* hemoglobin, *RBC* red blood count, *WBC* white blood cell count, *PLT* lymphocyte count, platelet, *ALB* albumin, *GLB* globulin, *TBIL* total bilirubin, *DBIL* direct bilirubin, *AST* aspartate aminotransferase, *ALT* alanine aminotransferase, *K*^*+*^ potassium, *PT* prothrombin time, *INR* international normalized ratio, *AFP* α-fetoprotein, *HAVF* hepatic arteriovenous fistula, *PVTT* portal vein tumor thrombus, *BCLC* Barcelona Clinic Liver Cancer, *ALBI* albumin–bilirubin, *C–P* Child–Pugh

**Table 3:** Multivariate analysis of potential predictive factors for non-response after TACE

Variable	Multivariate analysis	
	Adjusted OR (95% CI)	P value
DBIL	1.019 (0.984, 1.055)	0.2972
AST	1.003 (0.9983, 1.007)	0.2356
ALB	0.937 (0.893, 0.983)	0.0077*
Tumor size (cm)		
≤ 5	Reference	
> 5 and ≤ 10	1.731 (0.958, 3.128)	0.069
> 10	3.281 (1.694, 6.353)	< 0.001**
Numbers of tumor		
Single	Reference	
Multiple	1.340 (0.824, 2.179)	0.2378
HAVF		
No	Reference	
Yes	3.920 (1.639, 9.375)	0.0021**
PVTT		
No	Reference	
Yes	1.550 (0.92, 2.61)	0.0994
Integrality of tumor capsule		
Yes	Reference	
No	3.065 (1.627, 5.774)	< 0.001**

*TACE* transarterial chemoembolization, *OR* odds ratio, *DBIL* direct bilirubin, *AST* aspartate aminotransferase, *ALB* albumin, *HAVF* hepatic arteriovenous fistula, *PVTT* portal vein tumor thrombus

### Construction of the candidate predictive models

Based on the results from the multivariate logistic regression analysis, the ALB level, maximum tumor size, HAVF and integrality of the tumor capsule were used to develop the predictive model. The DBIL level is associated with liver function, which might affect the response to TACE. Therefore, the TBIL level was selected as a candidate predictive factor to construct the models. The candidate models are listed in Table 4. The GVIF of candidate predictive models and correlation analyses of predictors were performed to evaluate multicollinearity, and the results are shown in Supplemental Table 2 and Supplemental Figure 1. The value of GVIF was less than 1.5 in every model, and the correlation analysis indicated a weak correlation among those predictors.

**Table 4:** The candidate models for predicting non-respons after TACE

Model type	Intercept and variable	β	P value
Model1	Intercept	0.108	
	DBIL	0.021	0.230
	ALB	− 0.067	0.006
	Tumor size (cm)		
	≤ 5	Reference	
	> 5 and ≤ 10	0.707	0.018
	> 10	1.44	< 0.001
	HAVF		
	No	Reference	
	Yes	1.437	0.001
	Integrality of tumor capsule		
	Yes	Reference	
	No	1.36	< 0.001
Model2	Intercept	0.519	
	ALB	− 0.073	0.002
	Tumor size (cm)		
	≤ 5	Reference	
	> 5 and ≤ 10	0.698	0.019
	> 10	1.430	< 0.001
	HAVF		
	No	Reference	
	Yes	1.416	0.001
	Integrality of tumor capsule		
	Yes	Reference	
	No	1.372	< 0.001
Model3	Intercept	− 2.471	
	ALB (g/L)		
	≥ 35	Reference	
	< 35	0.731	0.004
	DBIL (μmol)		
	≤ 17	Reference	
	> 17	0.547	0.213
	Tumor size (cm)		
	≤ 5	Reference	
	> 5 and ≤ 10	0.735	0.014
	> 10	1.430	< 0.001
	HAVF		
	No	Reference	
	Yes	1.431	< 0.001
	Integrality of tumor capsule		
	Yes	Reference	
	No	1.344	< 0.001

*TACE* transarterial chemoembolization, *OR* odds ratio, *DBIL* direct bilirubin, *AST* aspartate aminotransferase, *ALB* albumin, *HAVF* hepatic arteriovenous fistula, *PVTT* portal vein tumor thrombus

### Evaluation and comparison of the performance of those candidate models and other staging systems

**Fig. 3:**
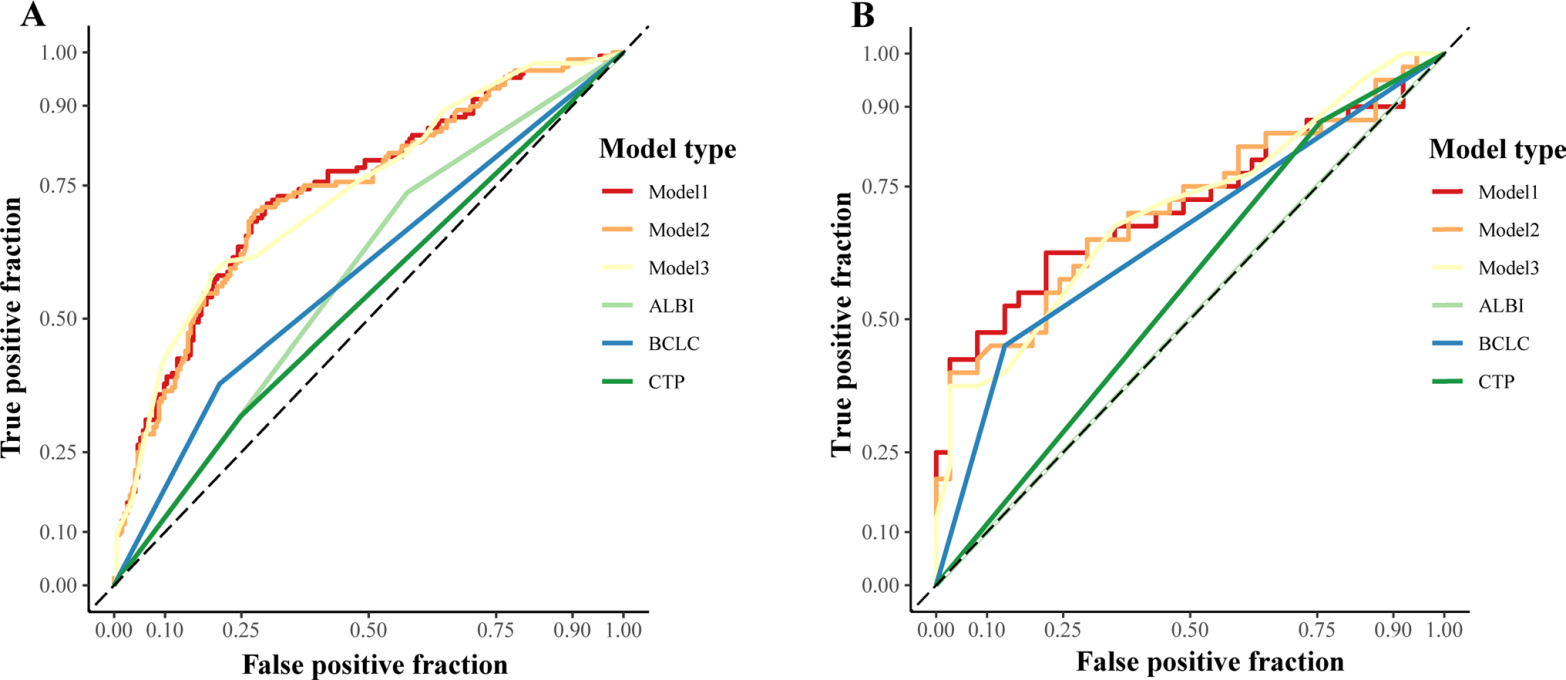
ROC curve analyses of candidate models and comparing the discrimination ability of BCLC staging, C–P class and ALBI grading in training group (**A**) and validation group (**B**). *ROC* receiver operating characteristic curve, *BCLC* Barcelona Clinic Liver Cancer, *ALBI* albumin–bilirubin, *C–P* Child–Pugh

**Fig. 4:**
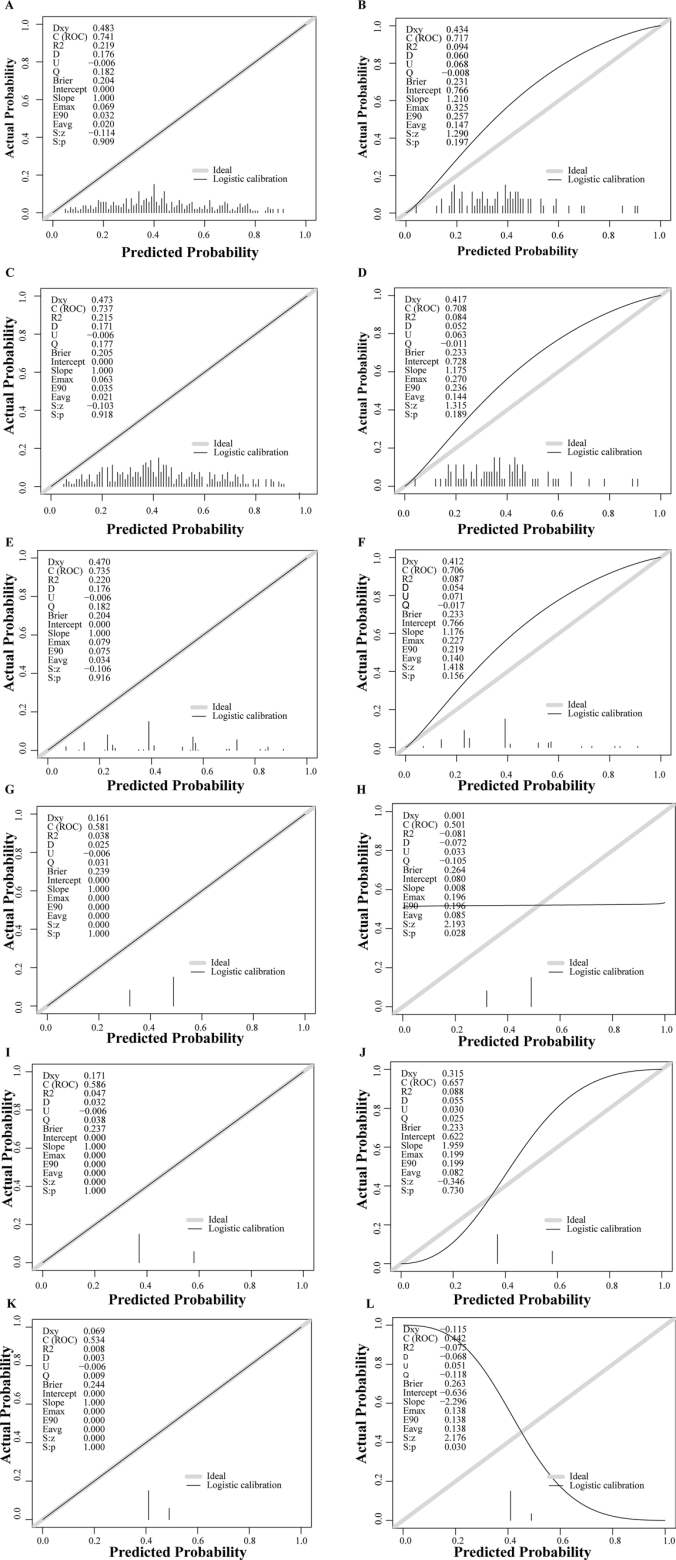
Calibration plot of candidate models, including model1 (**A**, **B**), model2 (**C**, **D**) and model3 (**E**, **F**), ALBI grading (**G**, **H**), BCLC staging (**I**, **J**) and C–P class (**K**, **L**) in training group and validation group, respectively. *BCLC* Barcelona Clinic Liver Cancer, *ALBI* albumin–bilirubin, *C–P* Child–Pugh

ROC curves of the candidate models, BCLC staging system, ALBI scoring system and C–P class are shown in Fig. 3A, B. The discrimination and calibration abilities of those models are shown in Table 5 and Fig. 4. The AUROCs of those candidate models were all greater than 0.7, indicating that those models had adequate discrimination ability in both the training and validation groups. Although the BCLC stage, C–P class and ALBI grade had modest discrimination and good calibration abilities for predicting the response to TACE in the training group, those staging systems showed poor calibration in the validation group. Continuous variables were converted to dichotomous variables, which might result in the loss of data, but the discrimination and calibration abilities of Model 3 was not worse than those of Model 1 and Model 2. Model 3 contained the variables of liver function (ALB and DBIL) and objective imaging characteristics (tumor size, HAVF, integrality of the tumor capsule) of the tumor. Therefore, Model 3 was named the T (**T**umor size) A (H**A**VF) C (integrality of the tumor **C**apsule) F (liver **F**unction) model. The AIC and the p value of the Hosmer‒Lemeshow test are shown in Supplemental Table 3 and Supplemental Table 4. Because the TACF model was more feasible than Model 1 and Model 2, we selected this model as the final model.

**Table 5:** Performance of candidate models and other staging score

Model type	Training group	Validation group
Model1		
AUROC	0.741	0.718
Calibration-in-large	0.069	0.325
Calibration slope	1.000	1.210
Model2		
AUROC	0.736	0.708
Calibration-in-large	0.063	0.270
Calibration slope	1.000	1.175
Model3		
AUROC	0.735	0.706
Calibration-in-large	0.079	0.227
Calibration slope	1.000	1.176
BCLC staging		
AUROC	0.586	0.657
Calibration-in-large	0.000	0.199
Calibration slope	1.000	1.959
C–P class		
AUROC	0.534	0.558
Calibration-in-large	0.000	0.138
Calibration slope	1.000	− 2.296
ALBI grading		
AUROC	0.581	0.501
Calibration-in-large	0.000	0.196
Calibration slope	1.000	0.008

*TACE* transarterial chemoembolization, *BCLC* Barcelona Clinic Liver Cancer, *ALBI* albumin–bilirubin, *C–P* Child–Pugh, *AUROC* area under the receiver operating characteristic

### Visualization of the model and clinical use

**Fig 5:**
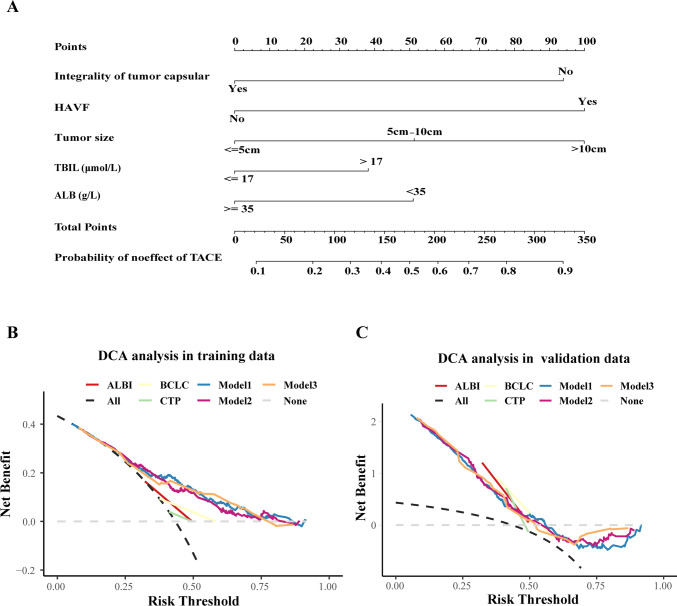
The nomogram of model3 (**A**) and the DCA curves of candidate models, BCLC staging, C–P class and ALBI grading in training group (**B**) and validation group (**C**). *BCLC* Barcelona Clinic Liver Cancer, *ALBI* albumin–bilirubin, *C–P* Child–Pugh

The nomogram of the final model is shown in Fig. 5A, and the exact scoring of the nomogram is shown in Supplemental Table 5. The results from the DCA of the candidate models and other staging systems are presented in Fig. 5B, C. In the training dataset, the DCA showed that for a risk threshold probability between 0.25 and 0.75, the final model obtained a greater net benefit than either the treat-all-patients or the treat-none scheme. However, in the validation group, the range of the risk threshold probability of the final model was narrower than that in the training group. However, the TACF model obtained a greater net benefit than the CLC stage, C–P class and ALBI grade.

According to the TACF model, the score for predicting nonresponse to TACE was categorized as low risk (less than 145), median risk (between 145 and 194) and high risk (greater than 194). An increasing trend for the risk was observed from the low-risk group to the high-risk group in both the training and validation sets (p value < 0.001 and p value = 0.001, respectively) (Table 6).

**Table 6:** Distribution of risk classification for predicting the score of non-effective TACE in training group and validation group

Risk category	Model score	Patients	Count of non-effective	Treatment recommendations
Training group				
Low risk	< 145	137	36	TACE
Median risk	Between 145 and 194	122	49	TACE + systemic treatment
High risk	> 194	82	63	Systemic treatment
Validation group				
Low risk	< 145	37	13	TACE
Median risk	Between 145 and 194	30	18	TACE + systemic treatment
High risk	> 194	10	9	Systemic treatment

*TACE* transarterial chemoembolization

In different time periods, the TACF model demonstrates excellent discrimination ability among HCC patients receiving TACE. The TACF model exhibits an AUROC value of 0.884 (Supplemental Figure 2), which consistently surpasses the BCLC staging (AUROC: 0.750), CTP staging (AUROC: 0.514), and ALBI staging (AUROC: 0.514). Furthermore, the TACF model also displays superior calibration ability compared to the BCLC staging, CTP staging, and ALBI staging (Supplemental Figure 3).

## Discussion

In the present study, we developed and validated simple and evidence-based TACF models incorporating objective imaging characteristics and individual liver function to stratify candidate patients with HCC into three risk categories before TACE. The TACF model showed an adequate discrimination ability, validation ability and clinical utility in both the training and validation groups. The features and novelties of this study are described below. (1) We designed and implemented the current study strictly based on the TRIPOD guidelines, and the sample size was adequate according to rigorous calculations. (2) We developed the first prognostic model, the results of which were obtained using manual calculation. (3) The three risk categories, which were divided by the TACF model, screened potential patients with HCC who would present a noneffective response to TACE.

The TACF model incorporated three objective imaging characteristics, including tumor size, HAVF, and integrality of the tumor capsule. The maximum diameter of the tumor size was proved that which were associated with OS and recurrence after surgical resection [3, 34–36]. In addition, it was also a risk factor for TACE refractoriness [37]. In our study, we divided the tumor size into three classifications, and it was associated with the first response to TACE, consistent with a previous study [21]. Numerous prior studies have identified the prognostic value of the number of tumors in patients with HCC receiving TACE [12, 14, 38]. However, the primary end outcome of those studies was OS or progression-free survival (PFS). However, in our study, the primary outcome focused on the first response to TACE. The univariate logistic regression analysis indicated that the number of tumors was a potential risk factor for nonresponse to TACE, but in the multivariate logistic regression analysis, the p value was not associated with the response to the first TACE. Kim et al. [9] found that multiple tumors were predictive factors for a failure to achieve a CR. The distinct point of our study compared to the previous study was that we combined CR and PR into one group, and we divided the multiple numbers of tumors into single and multiple tumors based on one lesion rather than three lesions. A similar result was reported in a study in which the tumor number was not a predictive factor for the first response to TACE [39]. Superselection of the tumor feeding artery was performed in every lesion, which contributed to the treatment response of every lesion being relatively independent. The predictive value of the number of tumors for the first response to TACE requires more research for confirmation. Hepatoportal arteriovenous fistula (HAVF) might redistribute the arterial flow into portal venous flow, which might weaken the first response and safety of TACE. In our study, HAVF was embolized before the iodized oil emulsion was injected to reduce the effect of TACE. However, the patients with HAVF tended to exhibit a poor response to TACE in our study, and another study also obtained a similar result [40]. Patients receiving TACE with an integrated tumor capsule had a better prognosis [41] and a machine-learning model to predict extrahepatic spread or vascular invasion also incorporated the tumor capsule [38]. The liver function of patients with HCC must be assessed to determine the treatment method. AST, ALB PT, and DBIL are serum biomarkers to reveal the liver status of patents with HCC. Therefore, we selected those indices for LASSO and logistic regression analyses. Finally, ALB and DBIL were selected for the construction of the TACF model. In our study, a low ALB level was associated with an unsatisfactory response to TACE, consistent with the results of previous studies [21, 42].

The assessment of liver function in patients with liver cancer often relies on the widely employed C–P class and ABIL score [28, 29]. The C–P score encompasses two subjective evaluation factors: hepatic encephalopathy and ascites severity. On the other hand, the calculation of the ABIL score criteria is relatively complex and requires the use of a calculator for its application. The AUROC values for both the C–P class and ABIL score were found to be below 0.6, with their calibration curves exhibiting inferior performance when compared to the TACF model. These outcomes imply that using liver function alone as a predictor for the effectiveness of initial TACE treatment may not yield satisfactory results. The BCLC staging system integrates liver function and tumor burden to categorize patients, displaying superior predictive efficacy for assessing the effectiveness of initial TACE treatment compared to relying solely on liver function scores, albeit lower than the TACF score. However, it is crucial to emphasize that the TACF score primarily focuses on patients undergoing their first TACE treatment, specifically evaluating outcomes following the initial TACE session. The evaluation of survival rates for patients receiving their first TACE treatment remains uncertain at present. Nevertheless, the TACF score demonstrates commendable discriminatory and calibration abilities in predicting the effectiveness of initial TACE treatment. In decision curve analysis, it exhibits higher net benefit values when contrasted with the C–P class, ABIL score, and BCLC staging system, implying that employing the TACF score as a guide for the initial treatment of liver cancer patients can yield greater clinical benefits. Significantly, in comparison to the Child–Pugh classification, ALBI score, and BCLC staging, the TACF staging system offers direct guidance for tailoring treatment strategies to potential TACE patients across various stages. This approach enables accurate treatment planning for individuals with advanced-stage liver cancer, utilizing widely accessible objective serum and imaging assessments.

Based on our calculations, for patients classified as intermediate risk after undergoing TACE treatment, we recommend considering adjunctive targeted therapy or immunotherapy. However, for high-risk patients, we advise against proceeding with TACE treatment and instead suggest focusing solely on systemic therapy. These recommendations aim to optimize the treatment approach based on the individual risk profile of each patient.

This study had several potential limitations. First, the TACF model was established based on a retrospective study. Second, although the patients were selected from two independent departments, the predictive model was developed and validated in one medical center, which might lead to the issue of the suitability of the TACF model for patients with intermediate-stage HCC before TACE who are treated at another medical center. Third, HBV infection is the main cause of HCC in China, but HCV infection and alcohol abuse are the main causes in other regions [24], which might result in different imaging characteristics of tumor. Thirdly, Despite the inclusion of variables like tumor size, HAVF, integrality of the tumor capsule, TBIL, and ALB in the TACF model, all of which are associated with the prognosis of liver cancer patients, this study did not consider patient survival as the primary outcome. As a result, the TACF model’s ability to forecast the survival duration of liver cancer patients has not been evaluated, underscoring the need for further investigation to assess its predictive efficacy in terms of patient survival. Fourthly, Due to the disparity in cost between drug-eluting beads TACE and conventional TACE, a significant proportion of patients at our medical center choose the more economical conventional TACE treatment. Therefore, the establishment of the TACF model is based on conventional TACE patients, and further research is needed to validate its applicability to drug-eluting beads TACE.

## Conclusions

We derived and validated a simple-to-use scoring model (TACF score) incorporating objective imaging characteristics and liver function to predict the response to the first TACE among patients with intermediate-stage HCC. The TACF score significantly classify patients into three risk groups according to the first response to TACE. The risk predictive model had adequate discrimination, validation and clinical utility and could be used to screen the appropriate patients with intermediate-stage HCC before TACE based on the individual response risk.

### Supplementary Information

Below is the link to the electronic supplementary material.

Supplementary file 1—Supplemental Figure 1: Correlation analysis among variables in candidate models. ALB: albumin; DBIL: direct bilirubin; HAVF: hepatic arteriovenous fistula. (TIF 1689 KB)

Supplementary file 2—Supplemental Figure 2: ROC curve analyses of candidate models and comparing the discrimination ability of BCLC staging, C–P class and ALBI grading in training group at different time periods. ROC: receiver operating characteristic curve; BCLC: Barcelona Clinic Liver Cancer; ALBI: albumin–bilirubin; C–P: Child–Pugh. (TIF 1409 KB)

Supplementary file 3—Supplemental Figure 3: Calibration plot of candidate models, including TACF model (A), BCLC staging (B), C–P class (C), ALBI grading (D) in training group at different time periods. BCLC: Barcelona Clinic Liver Cancer; ALBI: albumin–bilirubin; C–P: Child–Pugh. (TIF 3731 KB)

Supplementary file 4—Supplemental Figure 4: Information regarding missing values in overall (A), training (B) and validation group (C). TACE: transarterial chemoembolization; Hb: hemoglobin; RBC: red blood count; WBC: white blood cell count; PLT: platelet; ALB: albumin; GLB: globulin; TBIL: total bilirubin; DBIL: direct bilirubin; AST: aspartate aminotransferase; ALT: alanine aminotransferase; K^+^: potassium; PT: prothrombin time; INR: international normalized ratio; AFP: a-fetoprotein; HAVF: hepatic arteriovenous fistula; PVTT: portal vein tumor thrombus; BCLC: Barcelona Clinic Liver Cancer; ALBI: albumin–bilirubin; C–P: Child–Pugh. (TIF 4630 KB)

Supplementary file 5 (DOCX 44 KB)

## Data Availability

The original contributions presented in the study can be directed to the corresponding authors.

## Abbreviations

HCC: Hepatocellular carcinoma
TACE: Transarterial chemoembolization
LASSO: Least absolute shrinkage and selection operator
TRIPOD: Transparent Reporting of a Multivariable Prediction Model for Individual Prognosis or Diagnosis
BCLC: Barcelona Clinic Liver Cancer
ALBI: Albumin–bilirubin
C–P: Child–Pugh
Hb: Hemoglobin
RBC: Red blood count
WBC: White blood cell count
PLT: Platelet
ALB: Albumin
GLB: Globulin
TBIL: Total bilirubin
DBIL: Direct bilirubin
AST: Aspartate aminotransferase
ALT: Alanine aminotransferase
K^+^: Potassium
PT: Prothrombin time
INR: International normalized ratio
AFP: α-fetoprotein
HAVF: Hepatic arteriovenous fistula
PVTT: Portal vein tumor thrombus
mRECIST: Modified Response Evaluation Criteria in Solid Tumors
PD: Progressive disease
SD: Stable disease
CR: Complete response
PR: Partial response
DSA: Digital subtraction angiography
MRI: Magnetic resonance imaging
CT: Computed tomography
OS: Overall survival
GVIF: Generalized variance inflation factor
ROC: Receiver operating characteristic curve
AIC: Akaike information criterion
AUROC: Area under the receiver operating characteristic
DCA: Decision curve analysis
