# Supplementary figures and images for: Prediction model of no-response before the first transarterial chemoembolization for hepatocellular carcinoma: TACF score
Source: Discov Oncol. 2023 Oct 17;14:184. doi: 10.1007/s12672-023-00803-2 (PMC10581972; doi:10.1007/s12672-023-00803-2)

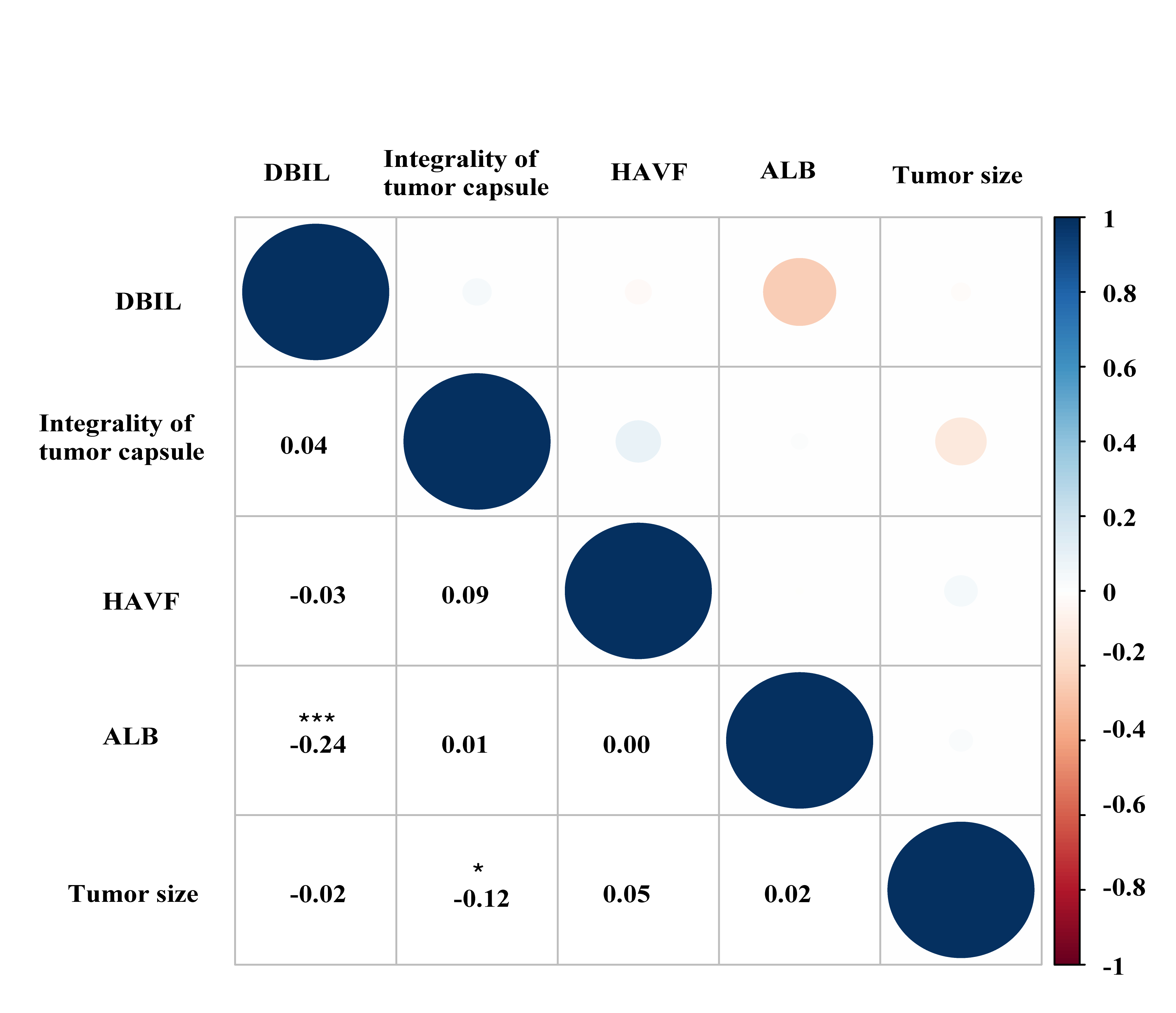

Supplement: Supplementary file 1 — Supplemental Figure 1: Correlation analysis among variables in candidate models. ALB: albumin; DBIL: direct bilirubin; HAVF: hepatic arteriovenous fistula. (TIF 1689 KB) [file 12672_2023_803_MOESM1_ESM.tif]

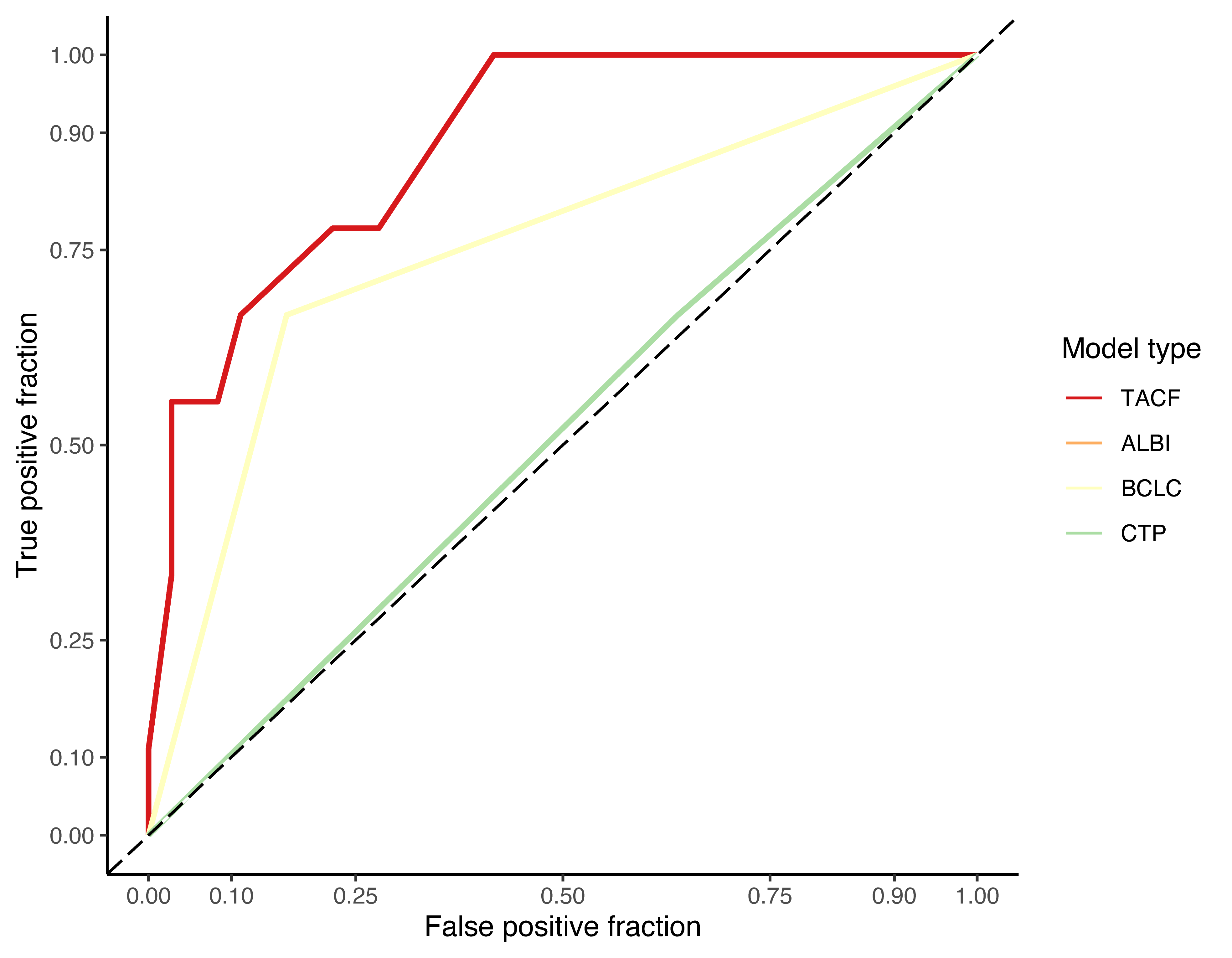

Supplement: Supplementary file 2 — Supplemental Figure 2: ROC curve analyses of candidate models and comparing the discrimination ability of BCLC staging, C–P class and ALBI grading in training group at different time periods. ROC: receiver operating characteristic curve; BCLC: Barcelona Clinic Liver Cancer; ALBI: albumin–bilirubin; C–P: Child–Pugh. (TIF 1409 KB) [file 12672_2023_803_MOESM2_ESM.tif]

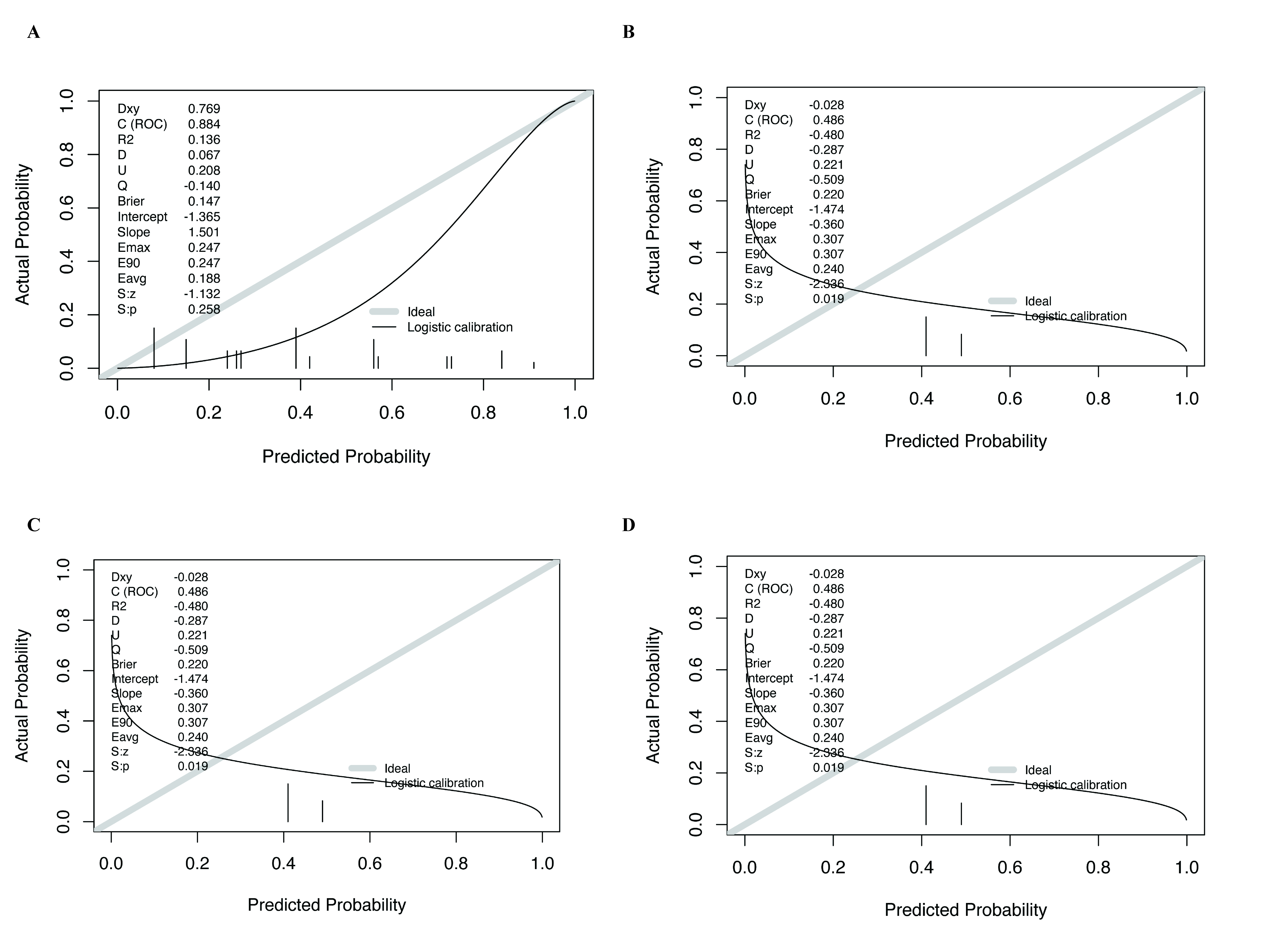

Supplement: Supplementary file 3 — Supplemental Figure 3: Calibration plot of candidate models, including TACF model (A), BCLC staging (B), C–P class (C), ALBI grading (D) in training group at different time periods. BCLC: Barcelona Clinic Liver Cancer; ALBI: albumin–bilirubin; C–P: Child–Pugh. (TIF 3731 KB) [file 12672_2023_803_MOESM3_ESM.tif]

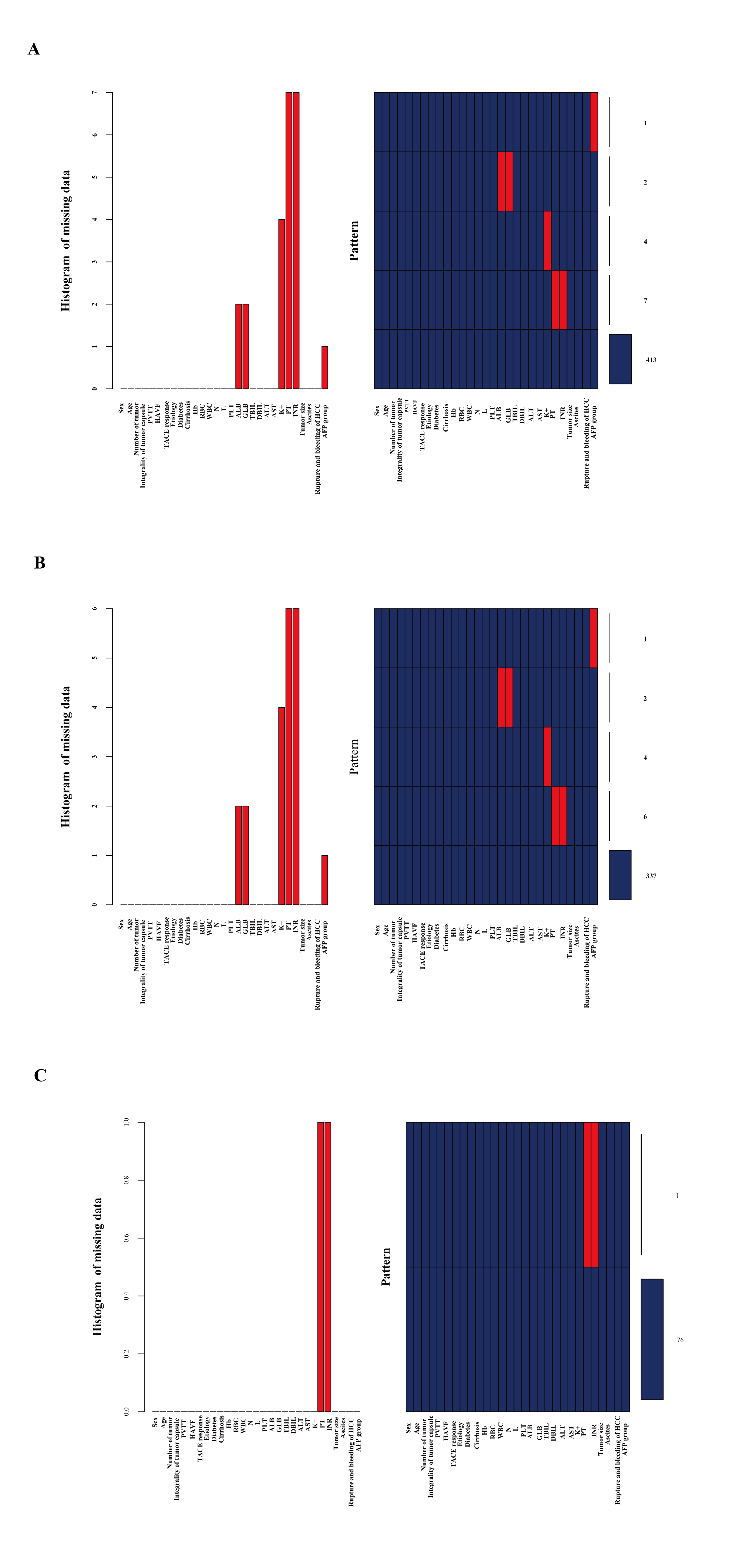

Supplement: Supplementary file 4 — Supplemental Figure 4: Information regarding missing values in overall (A), training (B) and validation group (C). TACE: transarterial chemoembolization; Hb: hemoglobin; RBC: red blood count; WBC: white blood cell count; PLT: platelet; ALB: albumin; GLB: globulin; TBIL: total bilirubin; DBIL: direct bilirubin; AST: aspartate aminotransferase; ALT: alanine aminotransferase; K+: potassium; PT: prothrombin time; INR: international normalized ratio; AFP: a-fetoprotein; HAVF: hepatic arteriovenous fistula; PVTT: portal vein tumor thrombus; BCLC: Barcelona Clinic Liver Cancer; ALBI: albumin–bilirubin; C–P: Child–Pugh. (TIF 4630 KB) [file 12672_2023_803_MOESM4_ESM.tif]
