# Supplementary material for: Prediction model of no-response before the first transarterial chemoembolization for hepatocellular carcinoma: TACF score
Source: Discov Oncol. 2023 Oct 17;14:184. doi: 10.1007/s12672-023-00803-2 (PMC10581972; doi:10.1007/s12672-023-00803-2)
Supplement: Supplementary file 5 — (DOCX 44 KB) [file 12672_2023_803_MOESM5_ESM.docx]

**Supplemental Table1** Checklist of Items to Developing or Validating a Multivariable Prediction Model for Diagnosis

| **﻿Section/Topic** | **﻿Item** | **﻿Development**  **or Validation?** | **﻿Checklist Item** |
| --- | --- | --- | --- |
| **﻿Title and abstract** |  |  |  |
| ﻿Title | 1 | D and V | The title identified the study as ﻿developing and ﻿validating a multivariable prediction model; the target population was the patients receiving the first TACE; the outcome was the response of TACE. |
| ﻿Abstract | 2 | D and V | The abstract had provided a summary of objectives, study design, setting, participants, sample size,  predictors, outcome, statistical analysis, results, and conclusions. |
| ﻿**Introduction** |  |  |  |
| ﻿Background and  objectives | 3a | D and V | The introduction explained the medical context as following: the model was prognostic mode; the rationale for developing ﻿ the multivariable prediction model and the existing models had been listed. |
|  | 3b | D and V | This study included development and validation of the model |
| ﻿**Methods** |  |  |  |
| ﻿Source of data | 4a | D and V | This study had described the study design and the source of data; the overall datasets was divided into development and validation datasets, randomly. Validation was also conducted on patients from different time periods. |
|  | 4b | D and V | This study had specified the start of accrual, end of accrual and of follow-up. |
| ﻿Participants | 5a | D and V | This study had described the study setting. |
|  | 5b | D and V | This study had described the eligibility criteria for patients. |
|  | 5c | D; V | This study had described the details of treatments received. |
| ﻿Outcome | 6a | D; V | This study had clearly defined the outcome. |
|  | 6b | D; V | This study was retrospective study. |
| ﻿Predictors | 7a | D; V | The study had ﻿clearly defined all predictors used in developing the multivariable prediction model. |
|  | 7b | D; V | This study was retrospective study. |
| ﻿Sample size | 8 | D; V | This study had explained the calculation of sample size |
| ﻿Missing data | 9 | D; V | This study had described how missing data were handled. |
| ﻿Statistical analysis  methods | 10a | D | This study had ﻿described how predictors were handled in the analyses. |
|  | 10b | D | ﻿This study had specified type of model, all model-building procedure |
|  | 10c | V | This study had ﻿described how the predictions were calculated |
|  | 10d | D; V | ﻿This study had specified all measures used to assess model performance and to compare multiple models. |
|  | 10e | V | This study did not describe ﻿any model updating. |
| ﻿Risk groups | 11 | D; V | This study provided the details on ﻿how risk groups were created. |
| ﻿Development vs.  validation | 12 | V | For validation, there was no difference from the development data in setting, ﻿eligibility, criteria, outcome, and predictors. |
| ﻿**Results** |  |  |  |
| ﻿Participants | 13a | D; V | A diagram had described the flow of participants through the study. |
|  | 13b | D; V | This study described the ﻿characteristics of the participants |
|  | 13c | V | The study ﻿showed a comparison with the development data of the distribution of important variables |
| ﻿Model development | 14a | D | This study ﻿specified the number of participants and outcome events in each analysis. |
|  | 14b | D | The study reported the unadjusted association between each candidate predictor and  outcome. |
| ﻿Model specification | 15a | D | This study present the full prediction model to allow predictions for individuals |
|  | 15b | D | This study ﻿explained how to use the prediction model. |
| ﻿Model performance | 16 | D; V | This study reported performance measures for the prediction model |
| ﻿Model updating | 17 | V | Not available. |
| ﻿**Discussion** |  |  |  |
| ﻿Limitations | 18 | D; V | This study discussed the ﻿limitations of the study. |
| ﻿Interpretation | 19a | V | ﻿For validation, this study discussed the results with reference to performance in the development data. |
|  | 19b | D; V | This study described the ﻿an overall interpretation of the results, considering objectives, limitations, results  from similar studies, and other relevant evidence. |
| ﻿Implications | 20 | D; V | This study discussed the potential clinical use of the model and implications for future research. |
| ﻿**Other information** |  |  |  |
| ﻿Supplementary  information | 21 | D; V | Not available. |
| ﻿Funding | 22 | D; V | This study described the ﻿source of funding and the role of the funders for the present study. |

**Supplemental Table 2.** The candidate models for predicting nonresponse after TACE

| Model type | Variables | GVIF |
| --- | --- | --- |
| **Model1** |  |  |
|  | DBIL | 1.059 |
|  | ALB | 1.082 |
|  | Tumor size | 1.122 |
|  | HAVF | 1.051 |
|  | Integrality of tumor capsule | 1.060 |
| **Model2** |  |  |
|  | ALB | 1.061 |
|  | Tumor size | 1.121 |
|  | HAVF | 1.050 |
|  | Integrality of tumor capsule | 1.061 |
| **Model3** |  |  |
|  | ALB group (cutoff = 35g/L) | 1.053 |
|  | DBIL group (cutoff = 17μmol) | 1.041 |
|  | Tumor size | 1.129 |
|  | HAVF | 1.055 |
|  | Integrality of tumor capsule | 1.059 |

TACE: transarterial chemoembolization; ALB: albumin; DBIL: direct bilirubin; HAVF: hepatic arteriovenous fistula.

**Supplemental Table 3.** **The AIC of those candidate models and other staging system**

| Model type | AIC |
| --- | --- |
| Model1 | 419.87 |
| Model2 | 419.31 |
| Model3 | 419.62 |
| BCLC stage | 458.70 |
| C-P class | 468.80 |
| ALBI grade | 461.09 |

AIC: Akaike information criterion; BCLC: ﻿Barcelona Clinic Liver Cancer; ALBI: albumin-bilirubin; C-P: Child-Pugh.

**Supplemental Table 4. The p value of Hosmer-Lemeshow of those candidate models and other staging system**

| Group | Model type | P value of Hosmer-Lemeshow |
| --- | --- | --- |
| Training group |  |  |
|  | Model1 | 0.401 |
|  | Model2 | 0.203 |
|  | Model3 | 0.059 |
|  | BCLC stage | 1 |
|  | C-P class | 1 |
|  | ALBI grade | 1 |
| Validation group |  |  |
|  | Model1 | 0.034 |
|  | Model2 | 0.156 |
|  | Model3 | 0.158 |
|  | BCLC stage | 0.859 |
|  | C-P class | 0.656 |
|  | ALBI grade | 0.770 |

BCLC: ﻿Barcelona Clinic Liver Cancer; ALBI: albumin-bilirubin; C-P: Child-Pugh.

**Supplemental Table 5.** Exact scores of all variables in nomogram

TACE: transarterial chemoembolization; HAVF: hepatic arteriovenous fistula; DBIL: direct bilirubin; ALB: albumin.

| **Variable** | **Nomogram Score** |
| --- | --- |
| **Tumor size (cm)** |  |
| ≤5 | 0 |
| > 5 and ≤ 10 | 51 |
| > 10 | 100 |
| **HAVF** |  |
| No | 0 |
| Yes | 100 |
| **Integrality of tumor capsule** |  |
| Yes | 0 |
| No | 94 |
| **DBIL (μmol)** |  |
| ≤ 17 | 0 |
| > 17 | 38 |
| **ALB (g/L)** |  |
| ≥ 35 | 0 |
| < 35 | 51 |
| **Total points** | **Probability of non-effect of TACE** |
| 22 | 0.1 |
| 78 | 0.2 |
| 116 | 0.3 |
| 147 | 0.4 |
| 175 | 0.5 |
| 203 | 0.6 |
| 234 | 0.7 |
| 272 | 0.8 |
| 329 | 0.9 |

**Supplemental Table 6.**

Demographics and clinical characteristics of patients with TACE at different time periods

|  | Training Group (n=350) | Validation Group (n=45) | P value |
| --- | --- | --- | --- |
| Sex |  |  | 0.809 |
| Male | 297 (84.9) | 37 (82.2) |  |
| Female | 53 (15.1) | 8 (17.8) |  |
| Age | 54.36 (12.71) | 58.07 (11.83) | 0.064 |
| Etiology |  |  | 0.217 |
| Other | 49 (14.0) | 10 (22.2) |  |
| HBV | 301 (86.0) | 35 (77.8) |  |
| Diabetes |  |  | 0.024 |
| No | 331 (94.6) | 38 (84.4) |  |
| Yes | 19 (5.4) | 7 (15.6) |  |
| Cirrhosis |  |  | 0.047 |
| No | 176 (50.3) | 15 (33.3) |  |
| Yes | 174 (49.7) | 30 (66.7) |  |
| Hb (g/L) | 128.0 [115.0, 142.0] | 122.00 [107.00, 135.00] | 0.035 |
| RBC (*10^12^/L) | 4.20 [3.76, 4.77] | 4.03 [3.52, 4.48] | 0.086 |
| WBC(*10^9^/L) | 5.30 [4.01, 7.29] | 5.40 [3.53, 8.76] | 0.404 |
| The count of neutrophils  (*10^9^/L) | 3.51 [2.42, 5.19] | 3.66 [2.50, 6.79] | 0.221 |
| lymphocyte count  (*10^9^/L) | 1.06 [0.78, 1.45] | 0.95 [0.71, 1.15] | 0.055 |
| PLT (*10^9^/L) | 147.00 [89.0, 207.75] | 148.00 [84.00, 227.00] | 0.799 |
| ALB (g/L) | 37.21 (5.13) | 37.42 (5.07) | 0.795 |
| GLB (g/L) | 29.90 [26.30, 33.90] | 31.80 [27.50, 36.20] | 0.046 |
| TBIL (μmol/L) | 15.30 [10.53, 20.58] | 18.20 [12.80, 25.20] | 0.010 |
| DBIL (μmol/L) | 5.70 [3.70, 8.80] | 4.60 [2.90, 6.90] | 0.046 |
| AST (U/L) | 37.50 [25.00, 57.75] | 34.00 [23.70, 53.90] | 0.980 |
| ALT (U/L) | 55.00 [38.00, 85.00] | 56.60 [40.10, 78.30] | 0.576 |
| PT (s) | 12.40 [11.70, 13.50] | 12.70 [11.80, 14.00] | 0.224 |
| K^+^(mmol/L) | 3.99 [3.70, 4.35] | 3.87 [3.51, 4.38] | 0.362 |
| INR | 1.10 [1.03, 1.20] | 1.12 [1.03, 1.22] | 0.831 |
| Ascites |  |  | 0.223 |
| None | 232 (66.3) | 25 (55.6) |  |
| Mild or Moderate | 88 (25.1) | 13 (28.9) |  |
| Severe | 30 (8.6) | 7 (15.6) |  |
| AFP (ng/ml) |  |  | 0.645 |
| < 40 | 116 (33.2) | 18 (40.0) |  |
| ≥ 40 and < 400 | 66 (18.9) | 7 (15.6) |  |
| ≥ 400 | 167 (47.9) | 20 (44.4) |  |
| TACE response |  |  | 0.004 |
| Responders | 196 (56.0) | 36 (80.0) |  |
| Non-responder | 154 (44.0) | 9 (20.0) |  |
| Tumor size (cm) |  |  | 0.753 |
| ≤5 | 107 (30.6) | 12 (26.7) |  |
| > 5 and ≤ 10 | 144 (41.1) | 18 (40.0) |  |
| > 10 | 99 (28.3) | 15 (33.3) |  |
| Numbers of tumor |  |  | 0.793 |
| Single | 175 (50.0%) | 24 (53.3) |  |
| Multiple | 175 (50%) | 21 (46.7) |  |
| HAVF |  |  | 0.378 |
| No | 315 (90.0) | 38 (74.4) |  |
| Yes | 35 (10) | 7 (15.6) |  |
| PVTT |  |  | 1.000 |
| No | 240 (68.6) | 31 (68.9) |  |
| Yes | 110 (31.4) | 14 (31.1) |  |
| Integrality of tumor capsule |  |  | 0.001 |
| Yes | 78 (22.3) | 24 （53.3） |  |
| No | 272 (77.7) | 21 （46.7） |  |
| Rupture and bleeding of HCC |  |  | 1.000 |
| No | 322 (92.0) | 41 （91.1） |  |
| Yes | 28 (8.0) | 4 （8.9） |  |
| C-P class |  |  | 0.370 |
| A | 246 (72.1) | 29 (64.4) |  |
| B or C | 95 (27.9) | 16 (35.6) |  |
| ALBI grade |  |  | 1.000 |
| I | 126 (36.2) | 16 (35.6) |  |
| II | 222 (63.8) | 29 (64.4) |  |
| BCLC stage |  |  | 1.000 |
| B stage | 94 (27.8) | 12 (26.7) |  |
| C stage | 249 (72.2) | 33 (73.3) |  |

TACE: transarterial chemoembolization; Hb: hemoglobin; RBC: red blood count; WBC: white blood cell count; PLT: lymphocyte count, platelet; ALB: albumin; GLB: globulin; TBIL: total bilirubin; DBIL: direct bilirubin; AST: ﻿aspartate aminotransferase; ALT: alanine aminotransferase; K^+^: potassium; PT: prothrombin time; INR: international normalized ratio; AFP: ﻿a-fetoprotein; HAVF: hepatic arteriovenous fistula; PVTT: portal vein tumor thrombus; BCLC: ﻿Barcelona Clinic Liver Cancer; ALBI: albumin-bilirubin; C-P: Child-Pugh.
